# Insurance Act Visitors

**Published:** 1914-07-11

**Authors:** 


### Insurance Act Visitors.

A GOOD deal of friction seems to be engendered in certain districts by the excess of zeal—misdirected, too, in many cases—of some of the "sick visitors" employed by approved societies. The proper function of these officials is to detect fraud upon the funds of the societies by those insured persons who are in receipt of sick benefit, although quite capable of following their employment. Primarily, of course, this is the business of the doctor, who certifies once a week whether or no the individual is fit for work. The "visitor's" function is to help the doctor by seeing the patient in his own home, by ascertaining whether the doctor's treatment is being properly carried out, and by checking the temptation to a convalescent but not fully recovered person of supplementing his sick benefit by doing odd jobs or other remunerated work. Such a visitor, when experienced, can often help the doctor to decide when or whether a given individual is fit to resume work. But the sole responsibility must rest upon the doctor.

It is not surprising, therefore, that doctors, nurses, and patients should resent an extension of the powers assumed by sick visitors, some of whom have shown a tendency to usurp the duties of the doctor by insisting on the return to work of insured persons. Instances are even vouched for in which professionally untrained sick visitors have presumed to examine cases, or even to remove surgical dressings from operation wounds with disastrous results. For this kind of interference the approved societies must be held responsible, for it is certain that no sick visitor would go to such lengths unless encouraged to do so by superior authority. Some of the societies, it is stated, are trying to enlist nurses to take up this sort of work. Unless it is made quite clear that diagnosis and the browbeating of the insured are no part of the duties of such sick visitors, no nurse or any other person should consent to accept such employment.

